# Return of the Megaureter: A Sequel no One Expected an Enigmatic Case of Ureteral Stump Syndrome

**DOI:** 10.1002/ccr3.72807

**Published:** 2026-06-04

**Authors:** Virginia Filippini, Giovanni Cochetti, Ambrosia Quaranta, Matteo Mearini, Francesco Valitutti

**Affiliations:** ^1^ Clinical Pediatrics, Department of Medicine and Surgery University of Perugia Perugia Italy; ^2^ Urology Unit, Department of Medicine and Surgery University of Perugia Perugia Italy

**Keywords:** abdominal pain, kidney, nephrectomy, subtotal ureterectomy, ureteral stump syndrome (USS)

## Abstract

This paper aims to emphasize the importance of not underestimating the potential complications of subtotal ureterectomy, despite their rarity. Ureteral Stump Syndrome can present with signs and symptoms that are not easily interpreted, and the range of differential diagnoses is broad and multidisciplinary.

A 14‐year‐old female patient presented with left flank pain and costovertebral angle tenderness. History‐wise the family reports that the girl underwent laparoscopic left ureter‐nephrectomy in 2017 in her country of origin but only partial clinical documentation was available. Eight years after surgery the patient experienced recurrence of left abdominal pain. At our hospital, first abdominal ultrasound confirmed ureteronephrectomy outcomes with no abnormalities detectable on ultrasound. In order to exclude a gynecological condition, an additional transabdominal ultrasound was performed and showed tubular formation firstly and erroneously attributable to transverse and descending colon (25 mm diameter). Due to the persistence of symptoms, abdominal MRI was performed which showed left megaureter (24 mm diameter) with high‐density fluid content and multi‐chambered abscess formation at the level of the left renal lodge (Figures 1, 2, 3, 4); in this context, the presence of residual calico‐pyelic cavities was also not excluded. Anterograde drainage was therefore positioned at the level of the left atrophic kidney in order to ensure the best removal of the abscess collection. Otherwise, urologists could have placed a double J ureteral stent, with the risk of less effective drainage. An early and effective removal of the abscess allowed systemic antibiotic therapy to obtain prompt improvement of her clinical conditions.

**FIGURE 1:**
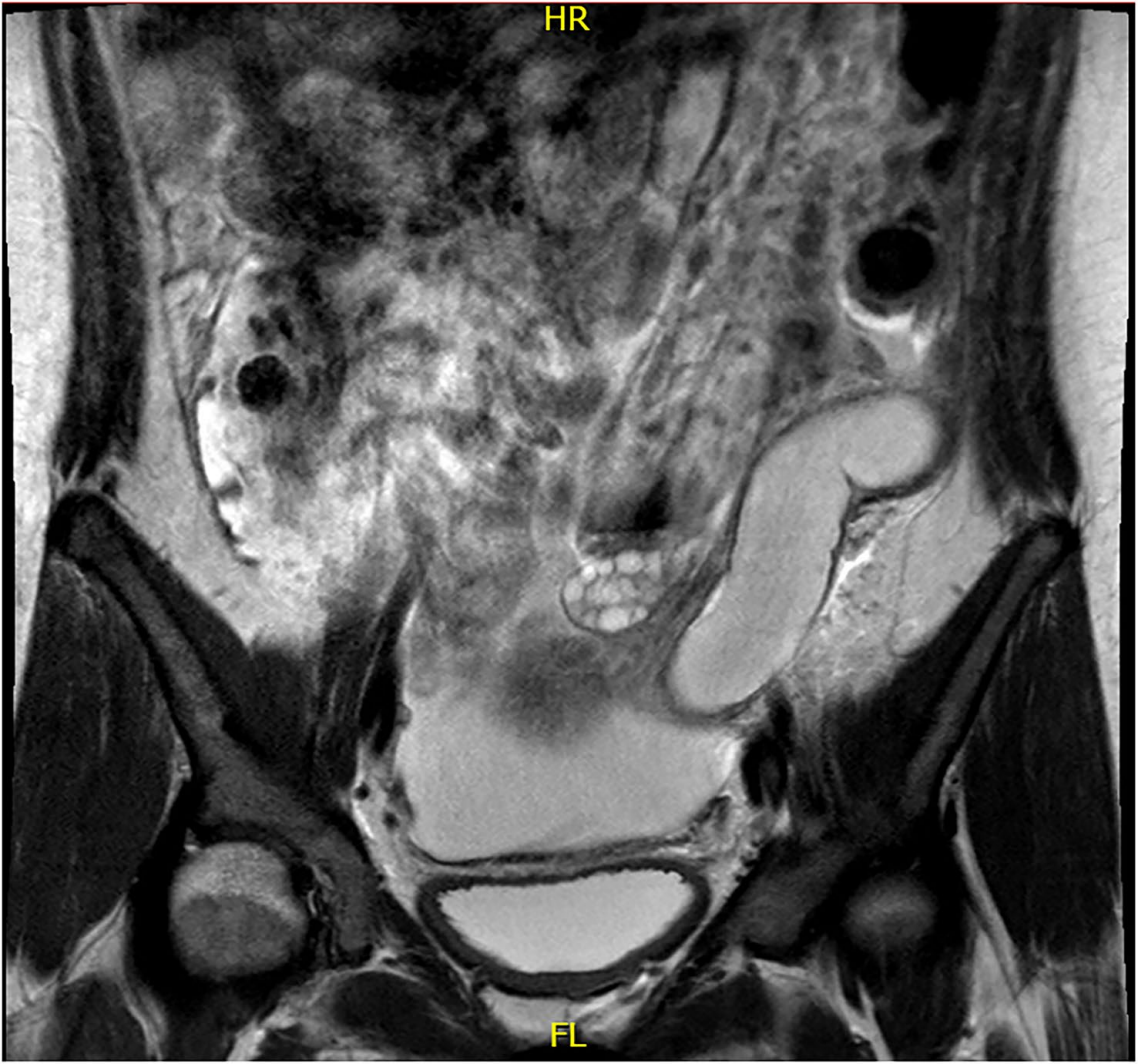
Multiparametric pelvic MRI, coronal T2W TSE HR sequence showing a partly tortuous tubular‐shaped structure in the left hemiabdomen compatible with ectatic left megaureter.

**FIGURE 2:**
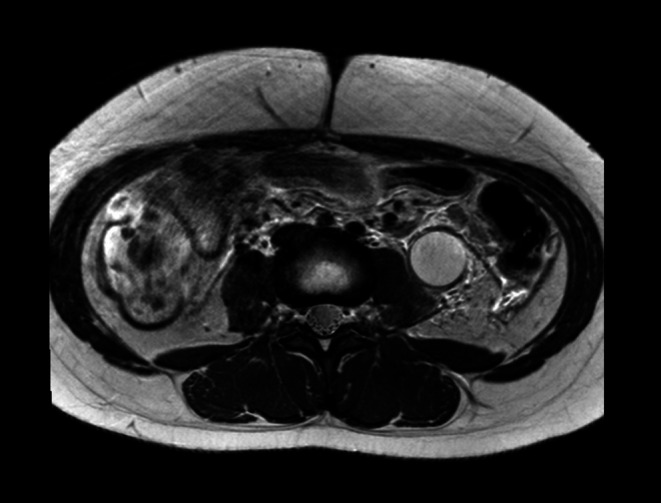
Multiparametric pelvic MRI, axial T2W TSE HR sequence showing ectatic left megaureter with diffusely overfluid contents and concentrically thickened and hyperintense walls.

**FIGURE 3:**
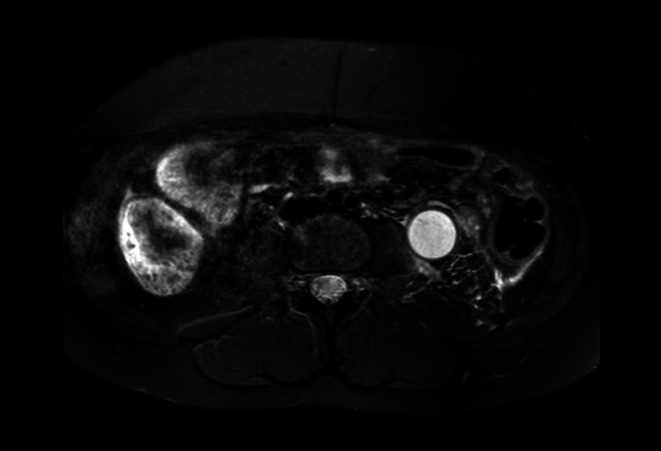
Multiparametric pelvic MRI, axial T2W SPIR HR sequence showing ectatic left megaureter with diffusely overfluid contents and concentrically thickened and hyperintense walls.

**FIGURE 4:**
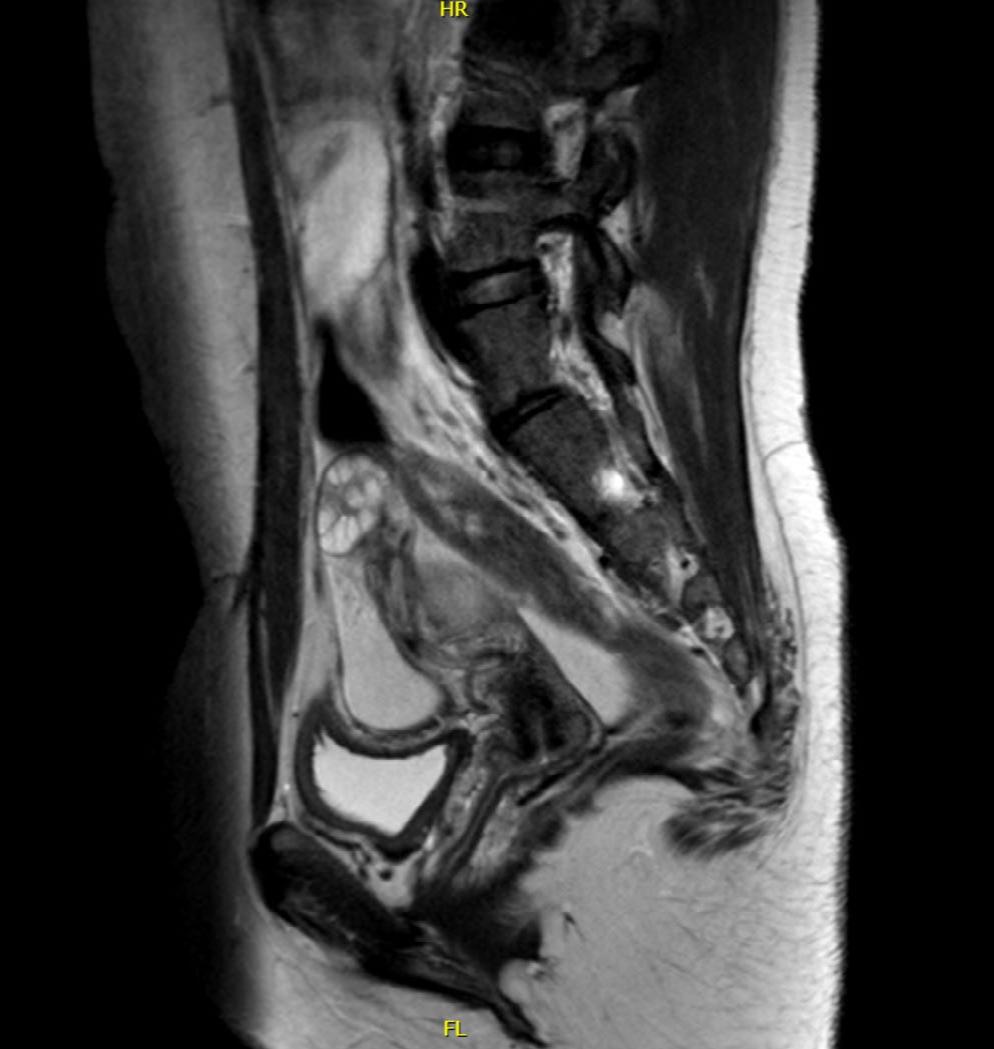
Multiparametric pelvic MRI, sagittal T2W TSE HR sequence. The most cranial part of the ureter in left renal lodge communicates with an oval component with irregular morphology and multiloculated thickened walls, compatible with pluriconchambered abscess formation.

The Ureteral Stump Syndrome (USS) is a rare complication that can occur after nephrectomy (incidence 0.8%–1%) [1]. During simple nephrectomy, two options can be considered: to perform a total ureterectomy concomitant with the nephrectomy, or to consider a subtotal ureterectomy. In most cases, the distal ureteral remnant is asymptomatic. Rarely, it could lead to USS, recurrent bacteriuria, stones, and even malignancy [1, 2, 3]. USS can clinically present with febrile urinary tract infections (UTIs), lower quadrant pain, hematuria and abscess [1]. The first step for the diagnosis is the collection of an accurate anamnesis and the observation of the clinical features, associated with the execution of the urinary tract ultrasound that usually allows to identify the dilated ureter residue. The possible presence of vesicoureteral reflux must be studied with voiding cystoureterography, then the abdominal MRI or CT must be performed to detect the presence of stones, abscess, empyema, masses or fistulous communications [1]. If symptomatic, the gold standard is surgical removal of distal ureteral stump by open or laparoscopic technique [1].

This paper aims to emphasize the importance of not underestimating the potential complications, despite their rarity, of subtotal ureterectomy. Ureteral Stump Syndrome can present with signs and symptoms that are not easily interpreted, and the range of differential diagnoses is broad and multidisciplinary.

## Author Contributions

**Virginia Filippini:** writing – original draft. **Giovanni Cochetti:** validation, visualization. **Ambrosia Quaranta:** writing – original draft. **Matteo Mearini:** visualization. **Francesco Valitutti:** data curation, project administration, supervision, validation.

## Funding

Open access funding of University of Perugia.

## Ethics Statement

Clinical case descriptions are clearly waived from our IRB approval when no change in current clinical practice is done.

## Consent

Both the patient and her parents signed an informed consent regarding the possibility of publishing the clinical case with deidentified data.

## Conflicts of Interest

The authors declare no conflicts of interest.

## Data Availability

The data that support the findings of this study are available on request from the corresponding author. The data are not publicly available due to privacy or ethical restrictions.
